# Effects of psychological ownership on teachers’ beliefs about a cloud-based virtual learning environment

**DOI:** 10.1186/s41039-018-0081-0

**Published:** 2018-08-30

**Authors:** Joanne Sau-Ching Yim, Priscilla Moses, Alia Azalea

**Affiliations:** 1 Universiti Tunku Abdul Rahman, Jalan Universiti Bandar Barat, 31900 Kampar, Perak Malaysia; 2 Tunku Abdul Rahman University College, Jalan Kolej, Taman Bandar Baru, 31900 Kampar, Perak Malaysia; 3 Faculty of Creative Industries, Universiti Tunku Abdul Rahman, Sungai Long Campus, Jalan Sungai Long, Bandar Sungai Long Cheras, 43000 Kajang, Selangor Malaysia; 4 Faculty of Arts and Social Science, Universiti Tunku Abdul Rahman, Kampar Campus, Jalan Universiti Bandar Barat, 31900 Kampar, Perak Malaysia

**Keywords:** Psychological ownership, Perceived usefulness, Perceived ease of use, Teachers’ experiences with VLE

## Abstract

Perceived usefulness and perceived ease of use constitute important belief factors when technology adoption decisions are made within a non-mandatory setting. This paper investigated the role played by psychological ownership in shaping teachers’ beliefs about using a cloud-based virtual learning environment (VLE). Psychological ownership is increasingly becoming a relevant phenomenon in technology adoption research, where people can feel psychologically attached to a particular technology. The study proposed that such phenomenon can also occur when using a VLE, and a hypothesised model with six constructs was tested with 629 Malaysian teachers from 21 schools. Results from structural equation modelling-partial least squares analysis found teachers’ experiences with the VLE significantly influenced psychological ownership, which in turn significantly predicted perceived usefulness and perceived ease of use of the VLE. Overall, the model possesses predictive relevance for the outcome predictors as indicated by Stone-Geisser’s *Q*^2^, and accounted for 61.6% of variance in perceived usefulness and 62.0% of variance in perceived ease of use. This study provides insights into the motivation behind teachers’ beliefs which are shaped by their experiences with the VLE. Implications for theory and practice were discussed based on the insights of the study.

## Introduction

It is important to understand teachers’ belief in technology use as it can affect their decision on its implementation in the classroom (Chien et al. 2014). Teachers are the frontliners in terms of integration of educational technologies in classroom as they mastermind the application of technological knowledge and pedagogical strategies using these technologies (Kong et al. 2017). In leveraging educational technologies to enhance teaching and learning, perceived usefulness and perceived ease of use are salient beliefs about technology which can affect users’ attitude and behaviour (Moses et al. 2013; Wang and Wang 2009). These beliefs have been found to be key determinants within the ambit of e-learning and learning management system (LMS), capable of explaining users’ acceptance, behavioural intention, continuance intention, and usage of these technologies (De Smet et al. 2012; Pynoo and van Braak 2014; Stantchev et al. 2014; Wu and Zhang 2014). They are central constructs to the Technology Acceptance Model (TAM), developed for research of technology used in utilitarian contexts (Davis 1989). Perceived usefulness is a driver for behavioural intention and attitude towards a particular technology, when individuals believe that using it would be advantageous for their job performance (Davis 1989). Perceived ease of use serves as an important intrinsic motivation for individuals to accept and use a particular technology. A technology that is perceived to be easy to use spares its users from the efforts of having to learn about it (Davis 1989). The efforts saved can be redeployed to the users’ core work tasks and increase their job efficiency and efficacy.

Much previous research had positioned perceived usefulness and perceived ease of use as predictors, rather than outcome variables (Teo 2011; Venkatesh 2000). There is a need to examine the antecedents of these perceptions, because they cannot entirely capture the subjective responses of its users (Rodríguez-Ardura and Meseguer-Artola 2016). As technology becomes ubiquitous in daily lives, psychological ownership has become a relevant phenomenon in technology adoption research (Klesel et al. 2016). Psychological ownership is the feeling of being psychologically attached to an object, where individuals feel as though an object’s ownership or a part of that object is “theirs” (Pierce et al. 2001). Research have linked and empirically supported the influence of psychological ownership on perceived usefulness and perceived ease of use (Barki et al. 2008; Paré et al. 2006; Yim et al. 2017). These studies reasoned that technology can become personally relevant to its users when they involve themselves in it, thereby inducing users’ feelings of ownership and buy-in for the particular technology. This notion is consistent with the theory of ownership which posits that individuals will have favourable perception towards owned objects compared to unowned ones (Pierce and Jussila 2011). Based on the potential influence of psychological ownership on users’ perceptions, this study aims to examine the development of perceived usefulness and perceived ease of use by focusing on the influence of ownership brought about by teachers’ experiences within a cloud-based VLE.

Cloud-based learning platforms are gaining popularity for its advantages in offering access to infinite on-demand resources, unlimited storage, and scalability in terms of bandwidth and computing functionalities (Stantchev et al. 2014). These advantages are not available in the conventional LMS that use grid-based computing technology such as Blackboard, Moodle, and WBLS. In this regard, services offered by cloud-based learning platforms are more advantageous than traditional LMS with benefits such as being “omnipresent” to the access of content with multiple devices, cloud storage, and provision of powerful collaborative support (Park and Ryoo 2013; Shiau and Chau 2016). A recent comparative research has suggested that users prefer the cloud computing platform rather than the traditional LMS in terms of platform usefulness and perceived ease of use (Stantchev et al. 2014). Despite its advantages, the current cloud-based learning platform in Malaysia, the Frog VLE, has received lukewarm application from teachers (Auditor General Report 2013).

The Frog VLE platform was implemented in 2012 as part of 1BestariNet (1SmartNet) project and made Malaysia the first nation in the world to connect all 10,000 public schools, 500,000 teachers, 5.5 million students, and 4.5 million parents using a single, cloud-based learning platform with high speed 4G internet connectivity (Hew and Syed Abdul Kadir 2016; Soon 2014). This VLE allows access to the schools’ community and parents, wherein teaching and learning can be conducted virtually, parents can view their children’s tests results and school news, while the school management can disseminate information via the platform (Soon 2014). It is integrated with educational applications such as Khan Academy and Google Apps for Education, providing users with an array of functionalities through widgets and built-in applications. Some of the functionalities include assignments, e-mail, booking calendar for school resources, creation of learning sites, quizzes, learning style reports, and Google Drive. There are also links to virtualised community and resources through forums, bookshelf, departmental sites for school subjects, the Pond, FrogStore, FrogAcademy, personal dashboard, and the school dashboard.

The first phase of implementation in 2013 to 2015 focused on infrastructural set-up and the equipment of teachers with VLE competency (Ministry of Education 2013). The second implementation phase which last from 2016 to 2020 concentrates on reviewing best practices using educational technologies for intervention of specific groups such as rural schools or under-enrolled schools (Ministry of Education 2013). As the VLE is at its second phase of implementation, investigating teachers’ experiences and their perceptions about it can help to inform its current state of implementation. This large-scale implementation of VLE offers an opportune time to examine teachers’ perceptions towards it, especially when there is a dearth of research on technology adoption among school teachers, with much of the previous studies focusing on students and university instructors (Hew and Syed Abdul Kadir 2016; Sanchez-Prieto et al. 2017).

### Literature review psychological ownership

The concept of psychological ownership originates from literature in philosophy, sociology, psychology, and human development, describing a possessive feeling of being psychologically attached to an object (Pierce et al. 2001). When psychological ownership is perceived, this possessive feeling can cause the object to become intertwined with the self, where an object becomes an extended self of the owner (Pierce and Jussila 2011). Within the work context, the potential targets of ownership can include tangible and intangible objects. Tangible objects can include one’s workspace, or work tools, whereas intangible objects may include the organisation itself or a project that one leads (Pierce and Jussila 2011). Technology can also be a target object for which psychological ownership can be developed, as evidenced by research carried out in contexts such as clinical information system (Barki et al. 2008), the virtual world (Lee and Chen 2011), and social media (Karahanna et al. 2015). Barki et al. (2008) defined psychological ownership in the technological context as the sense of ownership individuals feel for an information system (IS). The aforementioned study found psychological ownership to be prevalent among physicians who participated in developing a clinical IS, where ownership was developed for the IS and subsequently influence users’ beliefs about its usefulness and ease of use (Barki et al. 2008).

In comprehending the mechanism of psychological ownership, it is important to know the motivational bases behind the existence of psychological ownership. The theory proposed four needs that motivate ownership: to have a place, to have effectance, to be stimulated, and to have a self-identity (Pierce and Jussila 2011). These needs are not to be taken as the antecedents nor dimensions of psychological ownership, but they are important to address the questions of “why” psychological ownership come into existence (Pierce and Jussila 2011). The first need of having a place concerns individuals’ desire to possess a space that they feel comfortable. “To have effectance” reflects individuals need to be in control to satisfy the desire to be competent, where ownership arises due to the control exercise over an object. The third need of being stimulated pertains to human need for arousal, while having a self-identity refers the identification of oneself to a target object. In the current context of the study, a cloud-based VLE as a target of ownership can potentially fulfil these conditions for the development of psychological ownership. As VLE content and spaces are customised by teachers, they can feel psychologically attached to their ideas, design, and intellectual contribution. The personalised virtual spaces in the VLE can promote a sense of familiarity, satisfying the need of having a “place” (Pierce et al. 2001). By using and tailoring the applications in the VLE, teachers exercise control and discretion in using resources available satisfying the effectance need. As teachers involve with the VLE, they are stimulated with different functionalities and applications in the system, satisfying the need for arousal which can explain the dynamics of psychological ownership (Pierce and Jussila 2011). When teachers define VLE spaces according to their preferences, it can satisfy the need for self-identity as they express themselves through personalisation and customisation of the VLE. With this, the current study positions Frog VLE as a target of ownership for which feelings of psychological ownership can be developed by the teachers who use it.

Extant literature has identified three key experiences as antecedents that bring about psychological ownership of an object: (a) experienced control, (b) coming to know intimately, and (c) self-investment (Pierce et al. 2001; Brown et al. 2014; Zhao et al. 2016). *Experienced control* refers to a personal sense of control over a particular object experienced through its functional use, which give rise to a feeling of possessiveness. Control plays an important part on the semantics of ownership, and it is reasoned that objects that individuals exercise most control tend to be regarded as “theirs” (Pierce and Jussila 2011). Environments which afford individuals to experience control can foster the development of psychological ownership, as there are greater opportunities for exploration and to exercise discretion which satisfy the need to be effectance. C*oming to know intimately* about an object refers to the breadth and depth of knowledge an individual has about a target object. The more information acquired about an object, the more is known thoroughly about it. Hence, this experience promotes a sense of ownership as knowledge breeds familiarity, strengthen association, and increase proximity between a particular object and its owner (Brown et al. 2014; Pierce et al. 2001). *Self-investment* into an object refers to personal contribution invested into an object, such as one’s energy, time, imagination, and talents (Brown et al. 2014; Csikszentmihalyi and Rochberg-Halton 1981). By investing oneself into an object, individuals may consider the object to be part of themselves due to their personal contributions which emerged from the self.

**Fig. 1:**
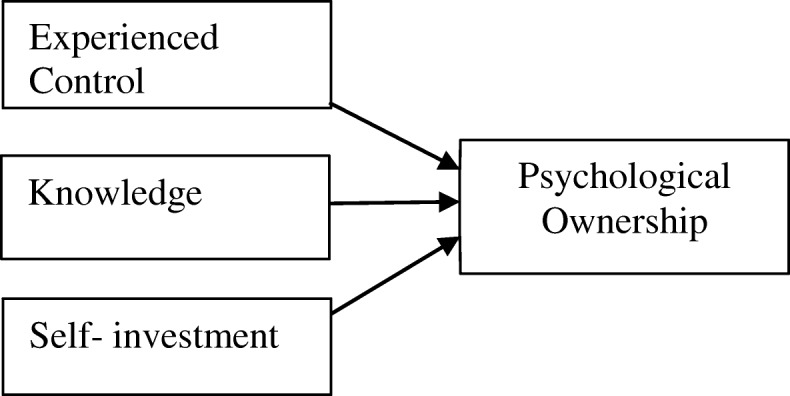
Psychological ownership and its antecedents

The potential of experienced control, coming to know intimately, and self-investment in fostering psychological ownership is well established across disciplines such as psychology, social psychology, human development, and sociology (Jussila et al. 2015). As psychological constructs are widely used in technology adoption research, psychological ownership had also been studied in different technological contexts and found to be a pertinent factor in affecting users’ perceptions and behaviour (Kirk and Swain 2018). These technologies include information systems, the social media, virtual world, online games, music-streaming applications, and virtual learning environments (Barki et al. 2008; Chen et al. 2016; Lee and Chen 2011; Moon et al. 2013; Sinclair and Tinson 2017; Zhao et al. 2016; Yim et al. 2017). It must be noted that psychological ownership is context-specific and not all studies have found these three key experiences to be its significant antecedents (Huang et al. 2016; Smith et al. 2014; Van Dyne and Pierce 2004). Hence, the present study attempts to test the basic tenets of psychological ownership and its antecedents in context of a cloud-based VLE as shown in Fig. 1.

### Perceived usefulness and perceived ease of use

The TAM is a theoretically justified and parsimonious model that makes a good ground theory for studying users’ beliefs in using LMS technologies (De Smet et al. 2012). The model is capable of explaining behaviour across a broad spectrum of end-users computing technologies with two key determinants that serve as motivation for technology use: perceived usefulness and perceived ease of use (Davis 1989). Perceived usefulness describes the belief that using a particular technology will improve job performance, while perceived ease of use refers to the belief that using a target technology will be free of effort (Davis 1989). Teachers will most likely use a technology if it is easy to use and its operation is free of effort (Moses et al. 2013). In the present study, perceived usefulness describes the degree to which teachers believe that using cloud-based VLE can improve their teaching performance, while perceived ease of use refers to the extent to which teachers believe such systems can be used easily (Wang and Wang 2009). As the Frog VLE is not a new technology and had been implemented earlier, perceived usefulness and perceived ease of use are grounded on actual experience of use and hence reflect the realised utility of the technology (Bhattacherjee 2001).

The importance of these beliefs in e-learning and LMS research has been affirmed with their causal influence on users’ attitude, satisfaction, system usage, intention to use, and continued intention to use (De Smet et al. 2012; Pynoo and van Braak 2014; Wu and Zhang 2014). In a study comparing six competing models to predict intention to use cloud computing classroom, the TAM exhibited the strongest effect size with perceived usefulness significantly influenced intention, and perceived ease of use significantly influenced attitude towards cloud computing classroom (Shiau and Chau 2016). In terms of LMS functionalities, De Smet et al. (2012) demonstrated that perceived usefulness and perceived ease of use are significant in influencing teachers to post announcements, upload exercises, receive students’ work, and publish documents in an LMS. In addition, perceived ease of use was also found to be a pre-cursor that give rise to teachers’ perception about LMS performance. In another study that investigated users’ motivations to replace traditional LMS tools with cloud-based services, perceived usefulness and perceived ease of use were found to be the key factors for evaluating cloud-based services (Stantchev et al. 2014). Hence, investigation of these beliefs can provide insights about the utility and functionality of a technology.

**Fig. 2:**
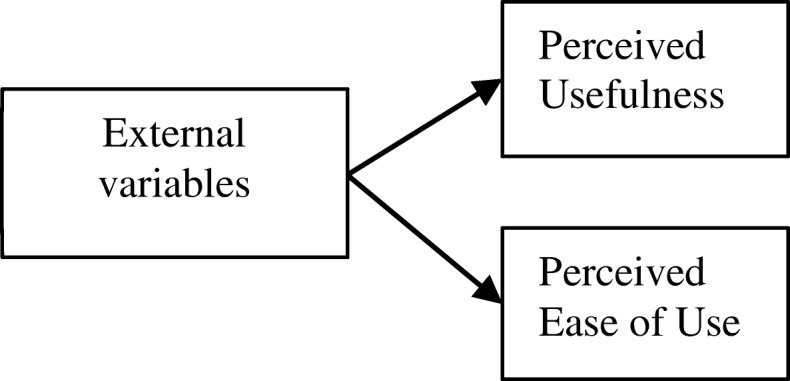
Effects of external variables on perceived usefulness and perceived ease of use

One of the short-comings of perceived usefulness and perceived ease of use was the absence of explanation for the motivations behind the reason for taking actions (Bagozzi 2007). TAM may be able to explain that teachers will use a VLE when they think it is useful and easy to use, but it does not contain motives to explain why they perceived it to be useful and easy to use. The original TAM had positioned system characteristics as predictors of perceived usefulness and perceived ease of use, but the model would not inform about the motivations that drive these two beliefs. As such, various external factors were incorporated to predict perceived usefulness and perceived ease of use in e-learning such as system quality, social motivations, self-efficacy, mobile anxiety, facilitating conditions, and course delivery as predictors of these beliefs (Sanchez-Prieto et al. 2017; Teo 2011; Wu and Zhang 2014). Despite the inclusion of various external variables, a recent review on TAM suggested there remain unexplored areas of the model’s application that can potentially enhance its predictive validity (Marangunić and Granić 2015). Figure 2 shows the relationship between external variables and perceived usefulness and perceived ease of use as posited by the TAM.

### Research model and hypotheses development

The study is focused on the central premise that teachers who use a cloud-based VLE may experience control over the VLE, acquire knowledge in using it, and invest aspects of themselves while implementing it. These experiences may elicit a sense of psychological ownership for the VLE which in turn influence their beliefs (perceived usefulness and perceived ease of use) about the VLE. With this, a hypothesised model was proposed with six constructs: experienced control, knowledge about VLE, self-investment into VLE, psychological ownership, perceived usefulness, and perceived ease of use.

### Experienced control and psychological ownership

*Experienced control* in this study refers to the control experienced by teachers when using a VLE. When control is continually exerted over an object, it can lead to a feeling of possessiveness and a sense of ownership towards the particular object (Brown et al. 2014). Control over a particular object is fundamental for it to be considered as part of the extended self (Belk 2013), and conditions which offer opportunities to exercise control will allow individuals to feel that they are the cause of their environment (Pierce and Jussila 2011). In a similar vein, the interactivity in cloud-based VLE provides varying degrees of control to users and can foster the emergence of psychological ownership for a particular technology. A VLE depends on teachers’ manipulation and control to create contents and activities, and teachers have a free-hand when using the VLE applications to create content that embodies their desired outcome. Empirical support for the influence of experienced control on psychological ownership has been established in the technological contexts such as the virtual world (Lee and Chen 2011), social media platform (Zhao et al. 2016), online games (Moon et al. 2013), and IS security (Huang et al. 2016). Hence, the following hypothesis is proposed to test its influence in the VLE context:

H1: Experienced control of VLE significantly and positively affects psychological ownership of VLE.

### Knowledge about a VLE and psychological ownership

*Knowledge about a VLE* pertains to the breadth and depth of information a teacher possesses about a VLE. When an object is manipulated to the extent that it is thoroughly known, the familiarity bred through such active association can increase proximity between an owner and the object (Brown et al. 2014; Pierce et al. 2001). This can occur in the current study because cloud-based VLEs are innovative learning environments that offer more comprehensive functionalities compared to traditional LMS systems. Teachers need to acquire knowledge and information about these functionalities to operate them effectively. Very often, this knowledge is accumulated through hands-on usage of the platform, staff development programmes, or information shared among peers. As teachers come to know the VLE better, the familiarity with it can facilitate the development of ownership for the VLE. A similar dynamic is found in the context of social media platform (Zhao et al. 2016), virtual world (Lee and Chen 2011), and music-streaming applications (Sinclair and Tinson 2017). Hence, the following hypotheses underpins the study:

H2: Knowledge about VLE significantly and positively affects psychological ownership of VLE.

### Self-investment and psychological ownership

Self-investment concerns the investment of personal aspects into an object that causes the object to flow from the self, fostering a sense of ownership for the object and self-identification of it (Brown et al. 2014; Paré et al. 2006). In this study, self-investment into a VLE refers to teachers’ contribution of themselves into using it such as their ideas, time, energy, efforts, and talents. Cloud-based VLEs offer virtual equivalents of conventional educational concepts, wherein teaching and learning can be conducted virtually. Teachers are very much self-directed in orchestrating activities in the VLE. Designing VLE contents is not the core of the teaching profession, and teachers need to devote more of themselves in activities to create learning sites, customise online assessment, and syntesise learning style reports. By investing their ideas, time, energy, and talents into the VLE, teachers may feel that the resultant contents reflect part of themselves, inducing ownership for the VLE. A similar mechanism is found in information system development, where users feel that the system flows from the self when they contribute their effort, skills, ideas, and attention into developing it (Barki et al. 2008; Paré et al. 2006). More recently, self-investment was also found to be the strongest predictor of psychological ownership in the social media context (Chen et al. 2016; Zhao et al. 2016). With this, the following hypothesis is formulated to investigate the influence self-investment in the study:

H3: Self-investment into VLE significantly and positively affects psychological ownership of VLE.

### Psychological ownership and user beliefs

Individuals will regard an object favourably when they perceive that the object belongs to them (Pierce et al. 2001). A sense of ownership can induce owners’ natural tendency to protect, enhance, or evaluate their possessions favourably (Van Dyne and Pierce 2004). Similarly, psychological ownership developed for a VLE can give rise to positive perceptions about the VLE. As suggested by previous research, a sense of ownership can result in the perception that a technology is useful and easy to use (Barki et al. 2008; Smith et al. 2014; Yim et al. 2017). In this vein, it is proposed that ownership developed for a VLE can help alleviate the efforts needed to operate it. In the context of this study, much effort is needed from teachers to set parameters to build and design their VLE environment, because Frog VLE is equipped with various customisable applications that run through the cloud. When the customised VLE become personally relevant and familiar to teachers, they may find it easy to operate and overcome technical barriers in using it. The following hypothesis is proposed for the effect of psychological ownership on perceived ease of use:

H4: Psychological ownership of VLE significantly and positively influences perceived ease of use.

Based on the preceding discussion, psychological ownership can also bring about positive perception about the usefulness of a technology (Barki et al. 2008; Smith et al. 2014; Yim et al. 2017). Psychological ownership taps into the psychological connection between owner and objects, and this connection can cause an object to be considered an extended self to its owner (Pierce and Jussila 2011). Consistent with the self-enhancement concept where individuals will strive to enhance their self-concept, individuals will have favourable opinion towards objects perceived to be part of themselves. It is possible for teachers to rate a VLE to be useful if it becomes a part of them. When ownership is perceived for a VLE, teachers may think that it is useful to facilitate their work, rather than add to it. Thus, the following hypothesis underpins this proposition:

H5: Psychological ownership of VLE significantly and positively influences perceived usefulness.

**Fig. 3:**
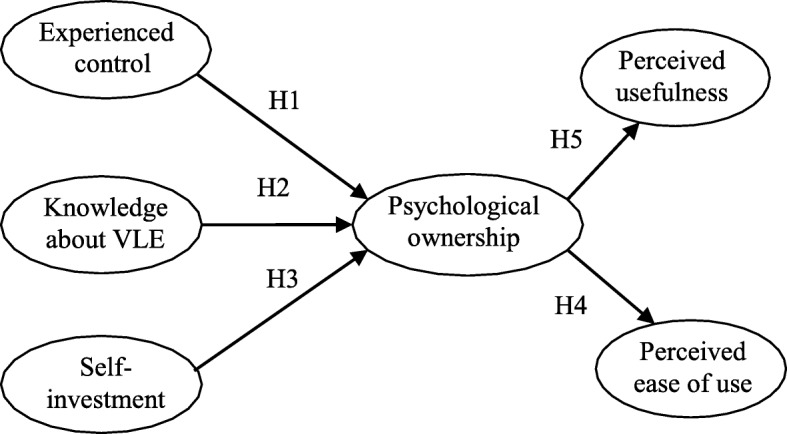
Research model

Figure 3 shows the conceptualised research model.

## Methods

### Design of the study

The present study entails the examination of a hypothesised model to explain perceptions about a VLE platform deployed to all teachers in public schools. It is therefore necessary to carry out a quantitative study with a sample that can provide sufficient power for statistical tests. As such, a survey method is used to collect data where questionnaires were filled-up by respondents who volunteered to participate in the study.

### Procedures and participants

Permission was sought from the Malaysian Ministry of Education to carry out research within two Malaysian states that consisted of 19 school districts. These districts were the cluster units where secondary teachers were sampled. With the permission, school principals were approached to distribute questionnaires to teachers. A study sample of 550 is required as calculated using G*Power 3.1 software with three predictors, to achieve 80% statistical at 0.05 alpha level and effect size (*f*^*2*^) of 0.02 (Faul et al. 2009). With this, 1000 questionnaires were distributed in anticipation of 50% response rate observed from local studies. A total of 660 questionnaires from 21 schools were returned where 629 were usable after screening for missing data, outliers, and unengaged responses. There were 488 females and 141 males, and teachers’ age ranged from 25 to 60, with a mean of 41 (SD = 8.49). Out of the 629 respondents, almost all of them (91.9%) had received training on the VLE. The average experience of using the system was 3.67 years (SD = 2.60), with usage experience ranging from half a year to 6 years. Only teachers who had used the system were included in the study, where respondents were filtered with a qualifying question that asked whether they had prior experience in using the system.

### Measures

Data were obtained through pencil-and-paper questionnaires that collected demographic information and the constructs’ measurement. The research adopted and adapted scales validated by published sources from the information system, educational technology, and psychological ownership domains (Brown et al. 2014; Barki et al. 2008; Davis 1989; Lee and Chen 2011; Wang and Wang 2009). Items were gauged with a 7-point Likert scale that anchored between one which indicated strongly disagree and seven which indicated strongly agree. Three subject matter experts reviewed the scales’ content suitability to ensure the items represent the intended area of investigation and match the underlying concepts of the constructs. As items in the adapted scales were presented in the English language, the instrument was translated to the Malay language using back-translation (Brislin 1970). Three translators were involved where the first one translated the questionnaire from the source language (English) to the Malay language. This version of the Malay language questionnaire was translated to English by the second translator who has no knowledge of the source items. Lastly, the third translator compared items from the source and back-translated questionnaire to identify concepts that are ambiguous, and discussions were held with the other two translators to revise the Malay version of the instrument. This method allowed the assessment of the quality of translation, as the instrument was subjected to three rounds of assessments with different translators. With this, the instrument was pre-tested with seven teachers to identify issues such as ambiguity in words used. After teachers’ feedback were obtained, the translators then decided on the most linguistically suitable translated words to use. A copy of the questionnaire with its referenced source is attached as Appendix. Additionally, a pilot test with 67 teachers was also conducted where satisfactory internal consistency was demonstrated with Cronbach alpha values as follows: experienced control of VLE (0.935), knowledge about VLE (0.946), self-investment into VLE (0.965), psychological ownership (0.890), perceived usefulness (0.967), and perceived ease of use (0.964).

## Results

### Data assessment for normality

Before proceeding to data analysis, the assumption of normality needs to be assessed to ensure the application of appropriate analysis procedures. Hence, data were subjected to multivariate skewness and kurtosis evaluation using an online software available at https://webpower.psychstat.org, where non-significance indicate adherence to normality assumption. However, results showed that data distributions were non-normal as indicated by Mardia’s multivariate skewness (*β* = 3.705, *p* < 0.001) and kurtosis (*β* = 68.076, *p* < 0.001). As such, the study employed the non-parametric Structural Equation Modelling analysis software SmartPLS 3.2.7 (Ringle et al. 2015) to analyse the data, and significance of path coefficients was tested with bootstrapping method of 5000 resamples. The study employed a two-stage analytical procedure (Anderson and Gerbing 1988), where the reliability and validity were assessed with a measurement model, and the proposed hypotheses were tested with a structural model.

### Assessment of measurement model

**Fig. 4:**
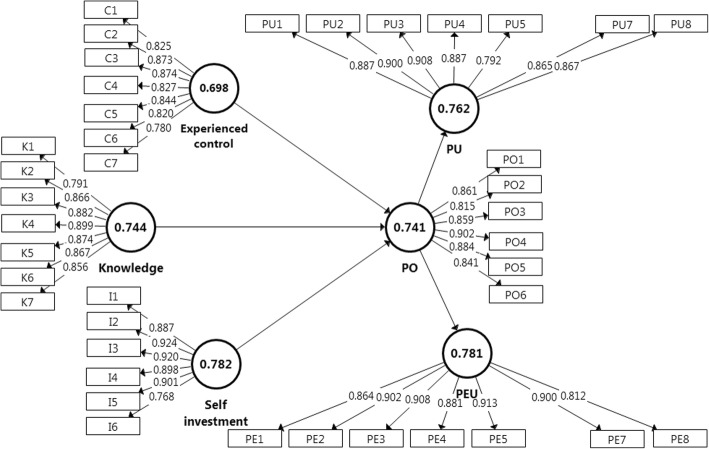
Measurement model with item loadings and AVE

The internal consistency of the constructs was assessed with Cronbach’s alpha (*α*) and Composite Reliability (CR), while convergent validity was established with average variance extracted (AVE). As shown in Table 1, internal consistency was verified with CR of 0.932 to 0.956 and Cronbach’s alpha values of 0.928 to 0.955 (Hair et al. 2017). Convergent validity was verified with AVE values of larger than 0.50, and all loadings of items measuring their designated constructs being more than 0.708 (Hair et al. 2017). To ensure discriminant validity of the constructs, inspection of cross loading found that two indicators from PEU were strongly correlated with items in PU. This resulted in the removal of one indicator from PEU and PU respectively because they could potentially affect the validity of results. After the removal, discriminant validity was verified with heterotrait-monotrait (HTMT) ratio with values lower than HTMT.85 that indicated that these constructs are distinctively different from one another (Henseler et al. 2015). Figure 4 shows the measurement model with indicator loadings and AVE for the constructs measured.

**Table 1:** Measurement model assessment

Constructs		Mean	Stand dev	*α*	CR	AVE	HTMT					
							1	2	3	4	5	6
1	EC	4.57	1.13	0.928	0.942	0.698						
2	K	4.15	1.18	0.942	0.953	0.744	0.687					
3	IN	3.74	1.22	0.944	0.946	0.782	0.604	0.848				
4	PO	4.29	1.16	0.930	0.932	0.741	0.657	0.731	0.766			
5	PEU	4.24	1.13	0.953	0.954	0.781	0.603	0.668	0.733	0.831		
6	PU	4.20	1.15	0.955	0.956	0.762	0.608	0.706	0.751	0.836	0.836	

Notes: *N* = 629. *EC* experienced control, *K* knowledge about VLE, *IN* self-investment, *PO* psychological ownership, *PEU* perceived ease of use, *PU* perceived usefulness

### Assessment of structural model

After the verification of measurement model, analysis proceeded to the next stage of structural model evaluation, where the path coefficient assessment was tested with bootstrapping of 5000 resamples. In testing the hypotheses, standardised path coefficients were analysed, where the significance of relationships was verified with three criteria: *t* values, *p* values, and the 95% confidence intervals which do not appear to be straddled in between a zero. In answering H1 to H3, psychological ownership was found to be significantly influenced by its antecedents of experienced control (*β* = 0.254, *p* < 0.001), knowledge about VLE (*β* = 0.170, *p* < 0.005), and self-investment (*β* = 0.439, *p* < 0.001). Results also showed that psychological ownership significantly influenced perceived ease of use (*β* = 0.788, *p* < 0.001) and perceived usefulness (*β* = 0.785, *p* < 0.001), supporting H4 and H5 respectively. Besides the evaluation of path coefficients which indicates the significance of the hypothesised relationships, the study also assessed the effect sizes of these relationships to evaluate the relative impact of an exogenous variable on an endogenous variable. The magnitude of the effect sizes (*f*^2^) was guided by the criteria of small = 0.02, medium = 0.15, and large = 0.35 (Cohen 1988). As shown from Table 2, the effect sizes of experienced control and knowledge on psychological ownership were small, while self-investment had a medium effect size on psychological ownership. Interestingly, psychological ownership has a large effect size on perceived usefulness and a nearing to large effect on perceived ease of use. Hence, all hypotheses were supported with defensible reliability and validity, supporting the study’s hypothesised model.

**Table 2:** Assessment of hypotheses

Hypotheses	*β*	Stand. error	*t* value	*p* value	Confidence interval	*f* ^2^	Supported?
H1 EC → PO	0.254**	0.041	6.208	0.000	[0.186, 0.320]	0.091	Yes
H2 K → PO	0.170**	0.059	2.901	0.003	[0.083, 0.274]	0.022	Yes
H3 IN → PO	0.439**	0.056	7.888	0.000	[0.341, 0.526]	0.166	Yes
H4 PO → PEU	0.788**	0.048	10.909	0.000	[0.441, 0.600]	0.340	Yes
H5 PO → PU	0.785**	0.045	12.169	0.000	[0.470, 0.618]	0.359	Yes

Note: *EC* experienced control, *K* knowledge about VLE, *IN* self-investment, *PO* psychological ownership, *PEU* perceived ease of use, *PU* perceived usefulness. **t value > 1.645, (one-tailed), *p* value < 0.005

**Fig. 5:**
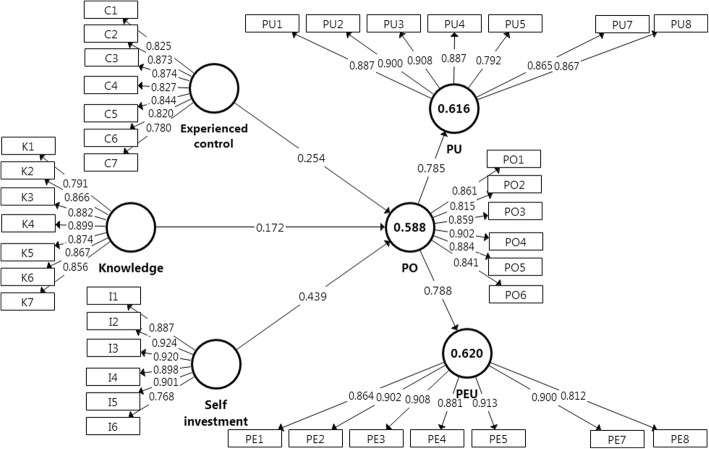
Structural model with path coefficients (*β*) and *R*^2^ for endogenous constructs

The predictive capability of the model is gauged, where it explained 58.8%, 61.6%, and 62.0% of variance in psychological ownership, perceived usefulness, and perceived ease of use respectively. These *R*^2^ values were above 26% which could be considered substantial (Cohen 1988). While *R*^*2*^ is useful for assessment of in-sample explanatory power, Stone-Geisser’s *Q*^2^ index was assessed to the measure the models’ out-of-sample predictive power. A *Q*^2^ value greater than 0 implies that the exogenous construct possesses predictive relevance in explaining their designated endogenous construct (Hair et al. 2017). As shown in Table 3, *Q*^2^ values for endogenous construct were greater than 0, establishing the capability of the study’s exogenous constructs in predicting psychological ownership, perceived ease of use, and perceived usefulness. The structural model consisted of the path coefficients and *R*^*2*^ is shown in Fig. 5.

**Table 3:** Coefficient of determination and Stone-Geisser’s index of endogenous constructs

Hypotheses	Coefficient of determination (*R*^*2*^)	Stone-Geisser’s (*Q*^2^)
Psychological ownership	0.588	0.407
Perceived ease of use	0.620	0.436
Perceived usefulness	0.616	0.450

## Discussion

The study aims to explain teachers’ perceived usefulness and perceived ease of use about a VLE with the concept of psychological ownership. Results demonstrated the significance of psychological ownership in the VLE context with all hypotheses supported. Overall, the model accounted for 61.6% of variance in perceived usefulness and 62.0% variance in perceived ease of use. Results also supported the proposition that psychological ownership of a VLE is influenced by experienced control, knowledge about the VLE, and self-investment into the VLE (H1, H2, H3). These findings were consistent with previous studies conducted in social media platforms (Chen et al. 2016; Zhao et al. 2016) and virtual communities (Lee and Suh 2015). Looking at the similarity between social media platforms and a cloud-based VLE, both platforms rely on the creation of contents as main activities. For instance, social media platforms are made of a coded infrastructure and the servers that host the contents, but it relies on users to create contents by uploading their posts or photos (Chen et al. 2016). Similarly, the cloud-based Frog VLE also relies on teachers to create learning sites and contents for different subjects facilitated by seamless access to resources with high degree of versatility (Soon 2014). It is reasonable to infer that psychological ownership is developed through these activities which allow teachers opportunities to exercise control, accumulate broad knowledge, and invest themselves. With regard to the similarity between a virtual community and a VLE, it is possible that teachers’ psychological ownership was significantly influenced by the control and autonomy they exercised in defining their virtual identity, where they can express themselves freely and engage in collaborative activities.

Self-investment into a VLE appeared to be the most important antecedent of psychological ownership, consistent with existing research (Brown et al. 2014; Lee and Chen 2011; Zhao et al. 2016). This suggests that the most profound means for teachers to develop psychological ownership is through the investment of their time, effort, energy, ideas, intellect, and talents in the VLE. When teachers invest their mental energy into creating learning contents, this form of investment is considered a form of digital cultivation that transforms digital products into meaningful possessions (Watkins et al. 2016). As teachers create their digital products, they may be involved in the ritualised practices of storing, saving, or backing-up their products for fear of losing them. These activities reinforces teachers’ possessive behaviour towards their VLE accounts, because it contains the digital products that they had spent much effort to create.

The study also found that psychological ownership developed for a VLE had positive significant influences on the VLE’s perceived usefulness with a large effect size and perceived ease of use with a nearing to large effect size (H4 and H5). The significance of the findings is consistent with existing research (Smith et al. 2014; Yim et al. 2017) which imply that psychological ownership can be valuable external variable in the TAM. The large effect size on perceived usefulness can serve the TAM well because research had shown perceived usefulness to be more important than perceived ease of use to effect user attitude and intention to use e-learning (Pynoo and van Braak 2014; Sanchez-Prieto et al. 2017; Shiau and Chau 2016). Results supported the notion that people evaluate objects more favourably if they feel a sense of ownership towards it (Van Dyne and Pierce 2004). In the context of the study, this implies that teachers who psychologically own their VLE tend to rate the platform favourably in terms of its usefulness and perceived ease of use. This is indicative of psychological ownership’s benefits in overcoming teachers’ perception of the complexity of cloud-based VLEs which offer more applications that run through the cloud compared to a traditional LMS. It is possible that increased psychological ownership can promote positive perception about the utility and functionality of cloud-based VLE among teachers.

### Implications of research

In terms of theoretical implications, the significance of psychological ownership on both key determinants of the TAM suggests that psychological ownership is a useful factor for a variety of theoretical perspectives. For instance, it can be viewed as a broader perspective of acceptance (Barki et al. 2008). Instead of explaining users’ acceptance or rejection of educational technology, psychological ownership describes how individuals can be intertwined with a particular technology until it become part of their extended self. This goes beyond willingness to use technology, encompassing a sense of possessiveness which implies personal involvement, empowerment, and attachment to the technology as a target of ownership. In addition, as the present study was conducted in the post-acceptance phase of technology, psychological ownership can be useful for examining users’ beliefs during the post-acceptance or post-adoption phase of technology. This construct can be useful for investigating systems that had been introduced earlier, because it is developed over time through experiences gained from using the system.

Recent research had suggested that Malaysian teachers are not maximising the adoption of technology in classrooms (Hew and Syed Abdul Kadir 2016), and psychological ownership can be useful for enhancing teachers’ beliefs about the usefulness and usability of these technology. The users’ experiences that underlie psychological ownership in this study namely experienced control, having knowledge, and self-investment are considered stable (Pierce and Jussila 2011; Karahanna et al. 2015), and understanding them help practitioners to identify the types of design and functionalities in cloud applications that need to be developed to facilitate ownership. As such, results of this study can have practical implications to assist in decision-making for resource allocations and policy strategies, and also contribute to the improvements of the VLE.

In terms of resource allocation, schools can be staffed with an IT personnel to assist teachers in overcoming difficulties in implementing the Frog VLE as such position does not exist in Malaysian schools. For system improvement, app developers can enhance control of features in VLE activities to enhance ownership. For instance, the versatility of the VLE can be enhanced by having more features for personalization instead of a few pre-determined themes for teachers to choose in developing their learning sites. This can result in a unique space that satisfies their needs of having their personal space and need for stimulation necessary for development of psychological ownership. In terms of policy strategies, teachers’ competency and knowledge about the VLE can be enhanced with a more inclusive approach of knowledge dissemination. Instead of the conventional top-down approach of trainings offered by the Malaysian ministry of education through seminars and conferences which cannot be inclusive, there is an increasing tendency for bottom-up approach which starts from the teacher themselves (Kong et al. 2017). For example, some countries in the Asian region have strategies for teacher development in e-learning such as peer-supported sharing of knowledge, engagement of teacher communities accountable for e-learning initiatives, involvement of universities to offer credit-bearing courses, and self-directed development via online credit-bearing courses (Kong et al. 2017). These strategies can be adapted in Malaysia as they are more inclusive to enable teachers to broaden their knowledge for effective usage of VLE. Moreover, self-initiated knowledge acquisition can strengthen ownership and buy-in of the VLE. Additionally, self-investment of time, ideas, effort, and talents was the most salient factor that results in psychological ownership for the VLE. Thus, the Ministry of Education can consider giving incentives to encourage teachers to contribute more of themselves into the VLE and participation in the VLE can be formalised for teachers, rather than on voluntary basis. Alternatively, cloud service providers may want to reward teachers based on their contribution into the VLE, such as giving recognition badges to acknowledge a particular level of involvement or offering discount on purchase of applications from the applications store.

## Conclusion

The feeling of ownership can be susceptible to corrosion, where owners perceive less of themselves in the target ownership (Watkins et al. 2016). Hence, it is fundamental that the experiences of control, knowing more about the VLE, and investing oneself into the VLE be sustained. As these experiences can be manipulated through system design and policy strategies, the study suggested approaches to enhance them to cultivate teachers’ long-term allegiance with a VLE. In spite of the significance reported for all hypothesised relationships, the current study has limitations which need to be addressed in future investigations. As psychological ownership is context-specific (Van Dyne and Pierce 2004), findings reported can only be generalised to the states’ locality from which data were obtained. The research model should be subject to further validation to enhance its external validity to support the inferences drawn, even though the quantitative evidence obtained demonstrated defensible validity and reliability. In short, this study provided evidence for future investigations to explore the theory of psychological ownership in educational technology research.

## Appendix

**Table 4:** Adapted items used to measure constructs

Source items	Adapted items	Referenced sources
Experienced control		
The influence over the things that affect the job.	I have influence over the things that affect my Frog VLE account.	Brown et al. (2014); Webster et al. (1993)
The influence over the tasks or parts of tasks.	I have influence over the tasks that I perform in my Frog VLE.	
The influence over job-related decisions made.	I influence decisions made on my Frog VLE.	
Setting own work deadlines.	I set my own deadlines in my Frog VLE.	
Control of the pace and scheduling of the work.	I control the scheduling of the tasks that involve Frog VLE.	
Control when using Lotus 1-2-3.	I felt in control when using the Frog VLE.	
Control over my interaction with Lotus 1-2-3.	I feel in control over my interaction with the Frog VLE.	
Knowledge about VLE		
I am intimately familiar with what is going on with regard to my job.	I am familiar with what is going on with regard to my Frog VLE account.	Brown et al. (2014); Zhang et al. (2011)
I have a depth of knowledge which relates to the job.	I have in-depth knowledge which relates to Frog VLE.	
I have a comprehensive understanding of the work that I am asked to do.	I have a comprehensive understanding of the functions in Frog VLE.	
I have a broad understanding of this job.	I have a broad understanding of Frog VLE.	
I always know where I can find the information I am looking for at Amazon.com’s website.	I always know how to find the information I need in Frog VLE.	
I visit Amazon.com very often.	I visit Frog VLE very often.	
I have been to Amazon.com many times.	I have visited Frog VLE many times.	
Self-investment		
I have invested a major part of “myself” into this job.	I have invested a major part of “myself” into Frog VLE.	Brown et al. (2014); Zhao et al. (2016)
I have invested many of my ideas into this job.	I have contributed many of my ideas into Frog VLE.	
I have invested a number of my talents into this job.	I have invested my talents into Frog VLE.	
I have invested a significant amount of my life into this job.	I have invested a significant amount of my time into Frog VLE.	
I often chat with friend using LINE.	I often perform work using Frog VLE.	
I often use LINE to know current status of friends	I often use Frog VLE for teaching and learning purposes.	
Psychological ownership of VLE		
I personally invested a lot in the implementation of the new system in my clinic.	I personally invested a lot in the implementation of the Frog VLE.	Barki et al. (2008)
When I think about it, I see a part of myself in the new system.	I can see a part of my teaching in the Frog VLE.	
I feel the new system belongs to all the doctors in my clinic.	I feel the Frog VLE belongs to all the teachers in my school.	
I feel a high level of ownership toward the new system.	I feel a high level of ownership toward the Frog VLE.	
I see myself as a champion of the new system in my clinic.	I see myself as a champion of the Frog VLE in my school.	
I configured the functionalities of the new system to better align it with my medical practice.	I organise the content of the Frog VLE to better align it with my teaching practice.	
Perceived usefulness		
Job performance	Using Frog VLE improves my job performance.	Davis (1989); Wang and Wang (2009)
Work more quickly	Frog VLE enables me to accomplish tasks more quickly.	
Increase productivity	Using Frog VLE increases my job productivity output.	
Effectiveness	Using Frog VLE improves my work effectiveness.	
Makes job easier	Using Frog VLE makes my job easier.	
Useful	I find Frog VLE useful to my job.	
Using web-based learning system enhances my interactions with the students.	Using Frog VLE enhances my interactions with students.	
Using web-based learning system increases the reuse rate of the course materials	Using Frog VLE increases the reuse rate of the teaching and learning materials.	
Perceived ease of use		
Easy to learn	It is easy for me to learn to use Frog VLE.	Davis (1989); Wang and Wang (2009)
Controllable	It is easy to get Frog VLE to do what I want it to do.	
Clear and understandable	It is easy for me to understand how to use Frog VLE.	
Flexible	The Frog VLE is flexible to work with.	
Easy to become skilful	It is easy for me to become skilful at using Frog VLE.	
Easy to use	Frog VLE is easy to use.	
I find it easy to get web-based learning system do what I want it to do corresponding to the ways I teach.	I find it easy to use Frog VLE to carry out my lesson plan.	
It is easy for me to recover from errors encountered while using web-based learning system.	It is easy for me to recover from errors encountered while using Frog VLE.	
